# Controlled infrared heating of an artic meadow: challenge in the vegetation establishment stage

**DOI:** 10.1186/s13007-019-0387-y

**Published:** 2019-01-19

**Authors:** Christophe Moni, Hanna Silvennoinen, Bruce A. Kimball, Erling Fjelldal, Marius Brenden, Ingunn Burud, Andreas Flø, Daniel P. Rasse

**Affiliations:** 1 Norwegian Institute for Bioeconomy Research – NIBIO, Høgskoleveien 7, 1430 Ås, Norway; 2 U.S. Arid-Land Agricultural Research Center, USDA, Agricultural Research Service, 21881 North Cardon Lane, Maricopa, AZ 85138 USA; 3 Norwegian Institute for Bioeconomy Research – NIBIO, Svanhovd, 9925 Svanvik, Norway; 4 Scanmatic Instrument Techonology AS (ITAS), Fredrik A. Dahls vei 20, 1430 Ås, Norway; 5 Department of Mathematical Sciences and Technology, Norwegian University of Life Sciences, 1432 Ås, Norway

**Keywords:** Climate change, Warming, Finnmark, Arctic, Infrared heaters, Meadow

## Abstract

**Background:**

Global warming is going to affect both agricultural production and carbon storage in soil worldwide. Given the complexity of the soil-plant-atmosphere continuum, in situ experiments of climate warming are necessary to predict responses of plants and emissions of greenhouse gases (GHG) from soils. Arrays of infrared (IR) heaters have been successfully applied in temperate and tropical agro-ecosystems to produce uniform and large increases in canopy surface temperature across research plots. Because this method had not yet been tested in the Arctic where consequences of global warming on GHG emission are expected to be largest, the objective of this work was to test hexagonal arrays of IR heaters to simulate a homogenous 3 °C warming of the surface, i.e. canopy and visible bare soil, of five 10.5-m^2^ plots in an Arctic meadow of northern Norway.

**Results:**

Our results show that the IR warming setup was able to simulate quite accurately the target + 3 °C, thereby enabling us to simulate the extension of the growing season. Meadow yield increased under warming but only through the lengthening of the growing season. Our research also suggests that, when investigating agricultural systems on the Arctic, it is important to start the warming after the vegetation is established,. Indeed, differential emergence of meadow plants impaired the homogeneity of the warming with patches of bare soil being up to 9.5 °C warmer than patches of vegetation. This created a pattern of soil crusting, which further induced spatial heterogeneity of the vegetation. However, in the Arctic these conditions are rather rare as the soil exposed by snow melt is often covered by a layer of senescent vegetation which shelters the soil from direct radiation.

**Conclusions:**

Consistent continuous warming can be obtained on average with IR systems in an Arctic meadow, but homogenous spatial distribution requires that the warming must start after canopy closure.

**Electronic supplementary material:**

The online version of this article (10.1186/s13007-019-0387-y) contains supplementary material, which is available to authorized users.

## Background

Global warming is going to affect agricultural production worldwide. Many of the highly productive agricultural regions in the world will be negatively impacted, with, for example, an expected yield reduction of 2% per decade in temperate regions in absence of adaptation [1]. However, certain colder climate regions might actually see their agricultural production increase with increasing temperatures [1]. This effect will be additional to that of CO_2_ fertilization, which has already resulted in a greening of many ecosystem types worldwide [2]. Regions that are now marginal in terms of their agricultural production because of high latitude could benefit most from climate warming. In a cold climate, warming is primarily expected to affect the ecosystem through the extension of the short growing season [3, 4]. Under high-latitude conditions, day-length changes very quickly in spring and fall. The effect of lengthening the growing season in such a high latitude is therefore quite uncertain. The Finnmark region of Norway is probably the most northern region in the world where agriculture is conducted. The Arctic is expected to experience highest climate warming [5]. Finnmark provides an Arctic end-member to understand the response of climate warming to high-latitude ecosystems, which are among the few expected to benefit from future warmer conditions.

Proper management of agricultural soils can help mitigate climate change through increased C sequestration and reduced N_2_O emissions [6]. This goal is reflected in the recent 4‰ initiative, which states that increasing soil C content by 4‰ annually would offset greenhouse gas emission worldwide. However, reaching this goal will be most difficult in high-latitude and high-C-content soils [7]. Will accelerated decomposition of soil organic matter under climate warming in high-latitude agricultural soils prevent further C storage? Or can this effect be compensated by increased C input from more productive plants? In situ experimental evidences is needed to answer these questions. Novel soil management methods will be needed to combat the negative effects of climate change on soil C and to further increase soil C sequestration. One of the key methods to reach these goals is biochar technology [6, 8]. Biochar is a pyrogenic C form that degrades at a much slower rate that other C forms in soil. Boreal and Arctic climates with cool summers and frozen soils during the winter are expected to further slow the degradation of these C forms. The effect of biochar on artic plant production and its degradation rate under normal and future-climate conditions are, therefore, important questions with respect to the possible future implementation of the technology in these regions.

Field experiments simulating future climate conditions are necessary to foresee actual ecosystem responses and provide the data needed to parametrize models of the terrestrial biosphere aiming at predicting ecosystem responses. One approach for conducting representative in situ warming experiments is the use hexagonal arrays of infrared heaters, which were specifically developed to produce a uniform distribution of the thermal radiation across large plots [9–12]. Such IR methods directly warm the land surface without warming the air first, and therefore they fail to reproduce a realistic vapor pressure difference between the leaves and the air [13]. However, they are still viewed as a realistic warming method that generates a low level of ecosystem disturbance provided that supplemental irrigation is employed [14, 15]. For this reason IR warming experiments have been set up in a wide range of environments [16], and their performance has been well documented in temperate regions [11, 12, 17–19]. However, although high latitude soils present the biggest threat for climate in terms of potential GHG emission [20, 21], high latitude ecosystems have rarely been subjected to IR warming experiments [16]. The few existing studies of this kind were conducted over small experimental plots and did not involve arrays of heaters [4, 22, 23]. Homogenous warming of large Arctic plots has never been attempted. Therefore, we set out to establish a controlled IR heating experiment at the Arctic meadow site of Svanhovd with the overall long-term project objective to determine effects on the carbon cycle, including changes in plant productivity, soil organic matter, and GHG emissions. The novel biochar management technique for soil C storage was also included for long evaluation. In the present study, we report on the initial phase of this project, where we developed and tested a controlled IR heating system installed at the establishment phase of a sown Arctic meadow. The objectives were to assess: (1) the homogeneity of the warming treatment on the exposed surface of canopy and soil, and (2) the further distribution of this warming within the soil as a function of depth, meadow management, and plant growth.

## Methods

### Experimental site

The experimental site was located in northern Norway in the Pasvik valley (69°27′13.50″ N; 30°02′09.82″E) at the border with Russia. The study site is a former fen that was drained for agricultural purposes in 1930’s and used as a meadow since then. It has been ploughed and reseeded every 6–8 years, as well as being fertilized annually either with manure or mineral fertilizers. Today the soil contains only 2% organic carbon (OC), so therefore it cannot be considered organic anymore, and it has become a mineral soil with silt loam texture and a stone content less than 0.1% [24].

### Experimental design

**Fig. 1:**
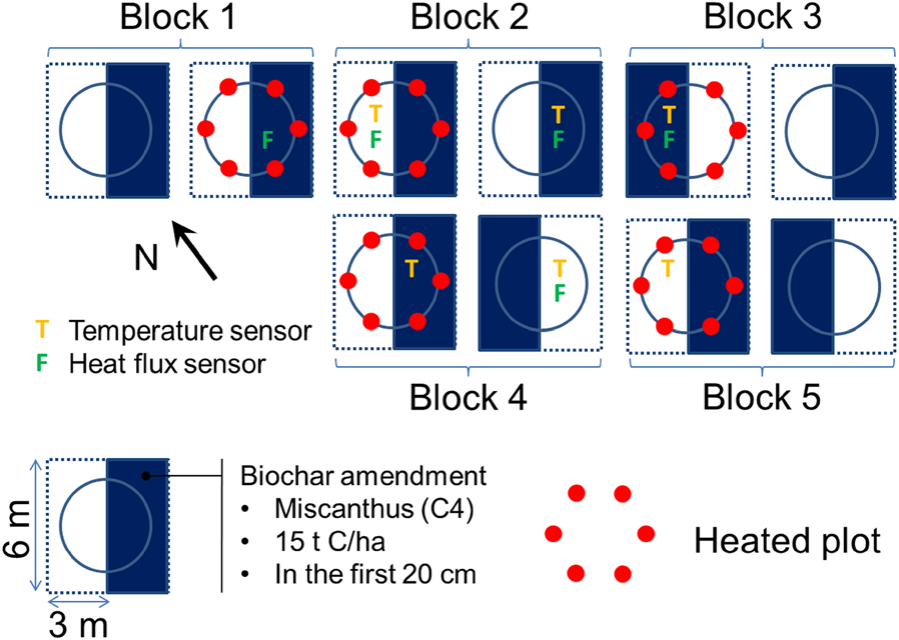
Experiment plot plan

Effects on a managed Arctic meadow of a simulated 3 °C warming and biochar application were tested with a split-plot design with 5 replicated blocks having warming as the whole plot treatment and biochar as the split-plot treatment. Each of the 5 blocks was composed of a warmed and a control whole plot (Fig. 1). In a first phase, 10 square plots (6 m by 6 m) were prepared so that half the surface of each plot was amended with biochar at a rate of 15 t C/ha. Plots were then rototilled to depth of 20 cm, while paying attention that no soil moved between the biochar and the control split-plots. In a second phase, circular bearing infrastructures of diameter 3.65 m and surface area 10.5 m^2^ were installed on all plots. Each of these infrastructures was equipped with either 6 heater casings or 6 dummies for warmed and control whole plots respectively (Additional file 1). The layout of heated/non-heated whole plots within each block as well as that of the biochar amended/non-amended sub-plots within each block was randomized (Fig. 1).

### Warming system

The warming system was adapted from Kimball et al. [9]. Each array was equipped with 18 heaters installed per group of 3 installed in 6 reflective housing distributed hexagonally along the circle. The heaters were Model FTE-1000 (1000 W, 240 V, 245-mm long by 60-mm wide) manufactured by Mor Electric Heating Assoc., Inc. Comstock Park, MI) mounted in Model ALEX-FFF reflective housings (756 mm long × 98.6 mm wide × 89.4 mm high). The 6 heating units were attached to a 3.65 m diameter ring whose height could be adjusted on the circular frame, so as to maintain a constant distance of 1.5 m between the heating units and the top of the canopy. Height adjustment was performed every 2 weeks to maintain the temperature treatment at the top of the canopy [25]. Each heating unit was tilted at an angle of 45 °C from horizontal toward the center of the plot. The height (0.4 times diameter) and tilt angle of the heating units were determined so as to maximize the theoretical thermal radiation distribution uniformity estimated through the angle factor (Additional files 1 and 2) [9]. For a height of 1.5 m and a tilt angle of 45 °C the relative range [(maximum–minimum)/mean)] and coefficient of variation (CV) of the theoretical thermal radiation distribution in a 3.4 m diameter surface located at the center of the heated zone were equal to 12% and 2.6% respectively, which is in the range of results obtained for different array configurations presented in Kimball et al. [9].

We built five similar hexagonal arrays of dummy heaters made from polystyrene foam to ensure that shading and other factors were as identical as possible between the heated and the reference plots.

Canopy surface temperatures in both control and warmed plots were sensed using a horizontal field of view infrared radiometer sensor (IRRs Horizontal; Model SI-1H1, Apogee Instruments, Logan, UT, USA), with individual calibrations.

The IRRs were deployed on one outer-ring post at the 2 m height, with the sensors pointed toward the center of the plot at an angle of 61° from horizontal (Additional files 1 and 4), so that the shape of the scanning window encompassed the largest part of a half-plot (Additional file 5). Since the modeled temperature distribution was symmetric to the center of the plot, the temperature measured on a half-plot should have been representative to that measured on a full plot.

Temperature regulation was achieved within each block by comparing the IRR signals of the non-heated and heated plots, and regulating power to reach the + 3 °C heating target for the temperature rise of the heated plots above the control plots day and night. 15-min averages of canopy temperatures were recorded. Signals from the IRRs over the heated plot were corrected for radiation from the heaters that was reflected from the vegetation in the 8–14 µm band [10]. The heaters were controlled using the PID control system with a data logger (Model CR1000, Campbell Scientific, Logan, UT, USA), as devised by Kimball [26]. The control system was hosted in a shed built at the experimental site. 15-min-average wind speed and wind direction were monitored on the site at 2 m height above the ground.

### Experimental site management

Plot preparation, including ploughing and biochar incorporation, was performed in August 2013. Experimental plots were plowed and seeded mid-May 2014 with a mixture of Arctic meadow plants including 53% timothy, 12% fescue, 20% bluegrass, 10% red clover, and 5% white clover (“Spire surfôr/beite Nord”). The warming system became fully operational May 5 (2014). The warming treatment started on June 5 (2014) and ended on September 29 (2014). Fertilizer (60 kg/ha, NPK 18-3-15, Yara) and herbicides (60 g/ha Gratil WG75 + 1 L/ha binding agent DP 0 + 250 L water/ha) were applied to the plots on June 6 and 23, respectively. Harvests were conducted three times during the growing season on July 4, September 2 and October 7. For the first harvest, temperature and moisture sensors were removed from the field during the operation and replaced afterward. For the two following harvests, the method was adapted to enable maintaining the sensors in the soil. To insure soil moisture remained well above the wilting point, the plots were irrigated with 3 cm of water on July 28 and 29 with a sprinkler.

### Monitoring

The experimental site was further monitored for soil temperature and heat flux. Six temperature sensors (Type-T, TC Ltd, UK) and 5 heat flux plates (model HFP01, Hukseflux Thermal Sensors, Delft, The Netherlands) were distributed in the experimental site, as evenly as possible, so that all treatments were monitored at least once for these three parameters (see Table 1 for sensor positions). All sensors were installed in the soil at the 7 cm depth and placed in the mid-point between the center of the plot and a heater-bearing pole. Soil temperatures were recorded on a 10 min basis, whereas heat fluxes were recorded every 30 min. Data recording was synchronized with measurements of surface temperature taken by the IRR and wind speed. Data were stored on the data loggers and collected 1 time per hour. LoggerNET data collection software from Campbell Scientific was used. Data are available online through Vista Data Vision hosted by ITAS (URL: http://graf.itasdata.no/download_data.php; username: Meadow; password: bioforsk0203).

**Table 1:** Number of sensor per treatments

Treatments	Plots	Sensor type	
		Temperature	Heat flux
Total	6	5	
Warming	Heated	4	3
	Control	2	2
Amendment	Biochar	3	3
	Control	3	2
Wrm × Am	Control–control	1	1
	Control–biochar	1	1
	Heated–control	2	1
	Heated–biochar	2	2

In addition to continuous monitoring, volumetric soil moisture of the surface layer (i.e. first 5 cm) was measured with a hand held time domain reflectometry (TDR) sensor (SM300, Delta-T device Ltd), 13 times between June 3 and August 8 (Table 2). For each date, measurements were taken at three random positions within each of the twenty split-plots. The soil moisture sensors were calibrated in the laboratory following the 2-points calibration procedure given by the manufacturer. Calibration was performed separately for soils amended and not amended with biochar, as biochar is known to affect the functioning of the TDR probes [27].

**Table 2:** Experimental site management practices

Date	Management
August 2013	Ploughing and biochar incorporation
15-05-2014	Seeding
20-05-2014	System turned on
05-06-2014	System functioning
06-06-2014	Fertilizer
23-06-2014	Herbicide
04-07-2014	Harvest 1
28-07-2014	Irrigation (3 cm)
29-07-2014	Irrigation (3 cm)
02-09-2014	Harvest 2
29-09-2014	System turned off
07-10-2014	Harvest 3

The uniformity of the warming treatment on the canopy or bare soil surfaces was checked for each plot on June 17 with a thermal imager (T640, FLIR) suspended by a crane over the heater array.

After each harvest, the collected biomass was dried in the oven at 60° for 3 days and weighed. Vegetation heights were measured 10 times between June 10 and August 19 (Table 3) within each split plot. They were determined from the average of 9 measurements taken in a dedicated 60 by 60 cm non-disturbed area. Coverage of plants was also visually estimated from this preserved area and extrapolated to the larger area. Plant coverage was also estimated through Normalized Difference Vegetation Index (NDVI) analyses of the plots using pictures taken with a modified RGB camera that captures Near Infrared (NIR) and Red (RED) Light. Briefly, pictures of the 20 split-plots were taken 22 times between June 27 and September 24. All pictures included a white reference to correct for varying light exposure between dates. For each pixel the NDVI was calculated with the following equation: NDVI = (NIR-RED)/(NIR + RED). Plant coverage was estimated as the proportion of pixels with a NDVI value larger than 1.4. By contrast, all pixels with a NDVI values less than 1.4 were considered to be bare soil.

**Table 3:** Measurement dates

Month	Surface soil moisture	Plant height and plant coverage
	Day number	Day number
June	3, 10, 16, 26, 30	10, 16, 26, 30
July	7, 14, 21, 29, 30	8, 15, 22
August	4, 12, 18	5, 11, 19

### Statistics

We used the lme4 package [28] of the R language for statistical analysis to perform a linear mixed effects analysis of the relationship between split-plot properties (i.e. soil moisture, vegetation height, harvest dry weight, and percentage of vegetation coverage) and the manipulated parameters of the experiment (i.e. Biochar and Warming). Effects of the treatments on surface soil moisture content were tested on the entire data set composed of 13 measurement dates, 3 measurements per split-plots, 2 split-plots per plot, 2 plots per block, and 5 blocks. Effects of treatment on plant heights were tested just before the first two harvests on June 30 and on August 19, respectively. Effects on harvested dry weight were tested for corresponding harvest dates on July 4 and September 2, respectively. Treatment effects on plant cover were tested just before the first harvest on June 30 and in the middle of the second growing period on July 22 as plant cover had reached 100% in every split-plot by the end of the second growing period. For fixed effects, we entered warming and biochar without interaction terms. For random effects, we had intercepts for date and split-plot nested in plot which in turn were nested in block when investigating soil moisture data, whereas for plant height, harvest dry weight and vegetation cover we took random intercepts for plot nested in block. P-values were obtained by likelihood ratio tests of model outputs obtained with versus without the tested effect. Model settings are summarized in Table 4.

**Table 4:** Linear mixed effect model characteristics

Dependent variable: X	Replicates	Time	Formula (function lmer − R package lme4)
Moisture	3	13 dates tested at once	X ~ Warming + biochar + (1|Block/Plot/Splitplot) + (1|time)
Height, harvest dry weight, plant coverage	1	2 dates tested individually	X ~ Warming + Biochar + (1|Block/Plot)

## Results

### Surface temperature difference per block between the heated and non-heated plots

**Fig. 2:**
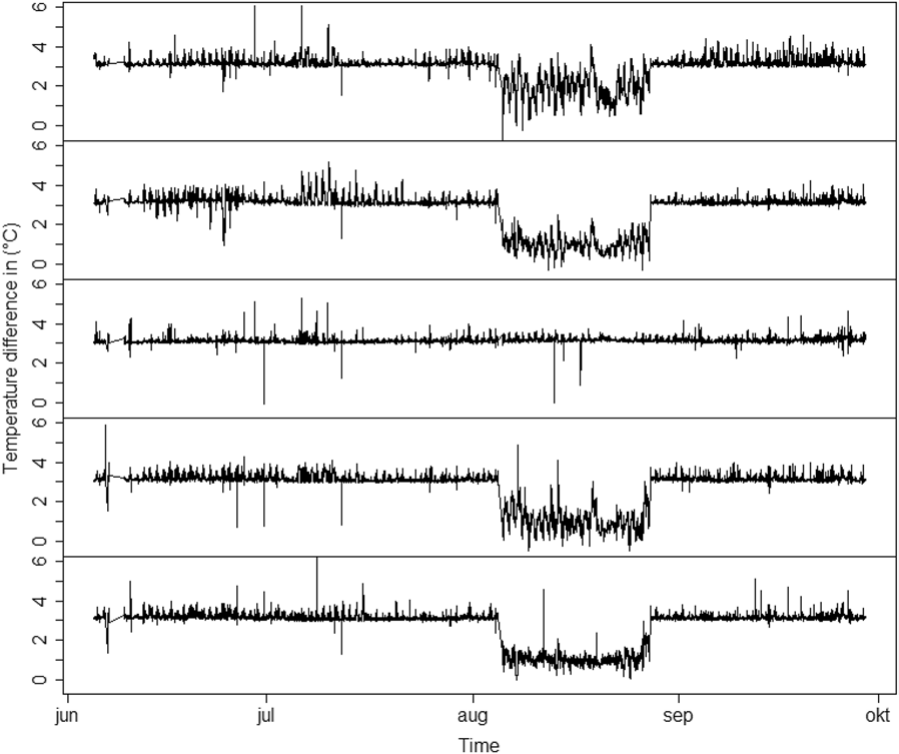
Temperature differences in surface between heated and non-heated plots from block 1 (top) to block 5 (bottom)

The warming system was functional from June 5 to September 29. During this period, differences of surface temperature recorded by the infrared radiometers (IRR) within blocks between heated and non-heated plots met the targeted 3 °C increase, except for a 20-day period from August 6 to 26 when temperature differences were lower in blocks 1, 2, 4 and 5 (Fig. 2). Electronic failures are current occurrence on the establishment year of such an experiment [29]. In our case, the cause was not identified.

**Fig. 3:**
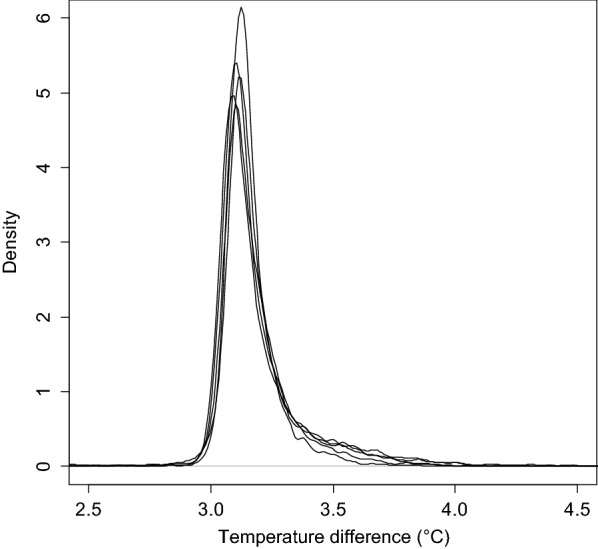
Temperature difference distributions between heated and non-heated plots per block for the normally functioning period (from June 5 to August 6 and from August 26 to September 29)

During the normal functioning period, the temperature difference distribution was positively skewed, with highest data frequency slightly above 3 °C and a tailing off that was close to zero at 4 °C (Fig. 3). Because the distribution was skewed, the median was preferred to the mean to estimate the position of the mode. The 5-block average of the median value of temperature increase was 3.13 ± 0.01 °C, indicating that the temperature control system performed according to target specifications and generated highly repeatable warming patterns between blocks.

**Fig. 4:**
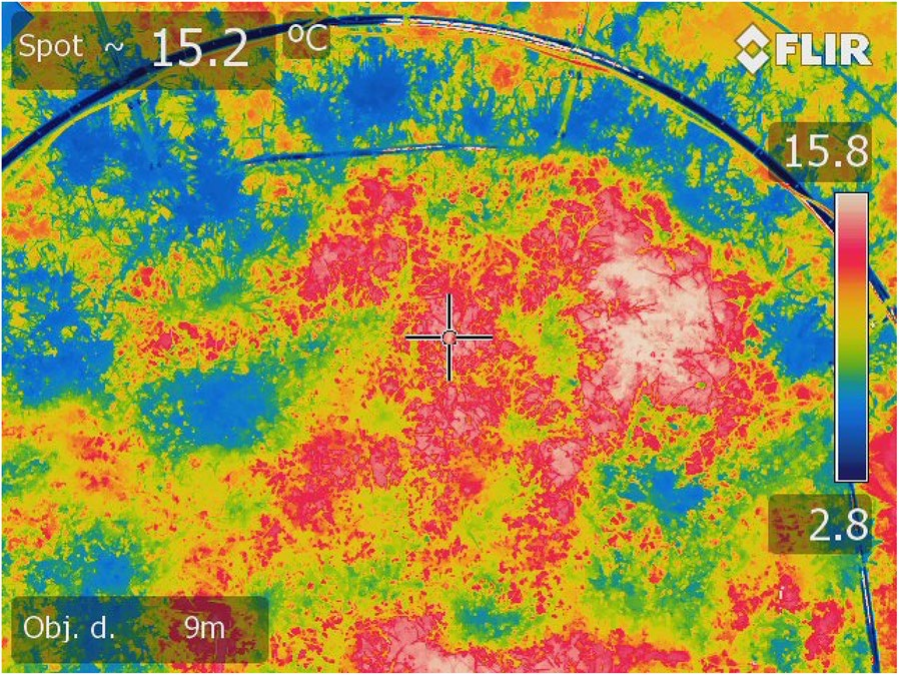
Thermal image of the distribution of warming on a warmed plot

Thermal imaging showed that within the warmed plots the surface temperature was heterogeneous ranging between 6.3 and 15.8 °C for the patches of vegetation and bare soil, respectively (Fig. 4).

### Soil moisture

**Fig. 5:**
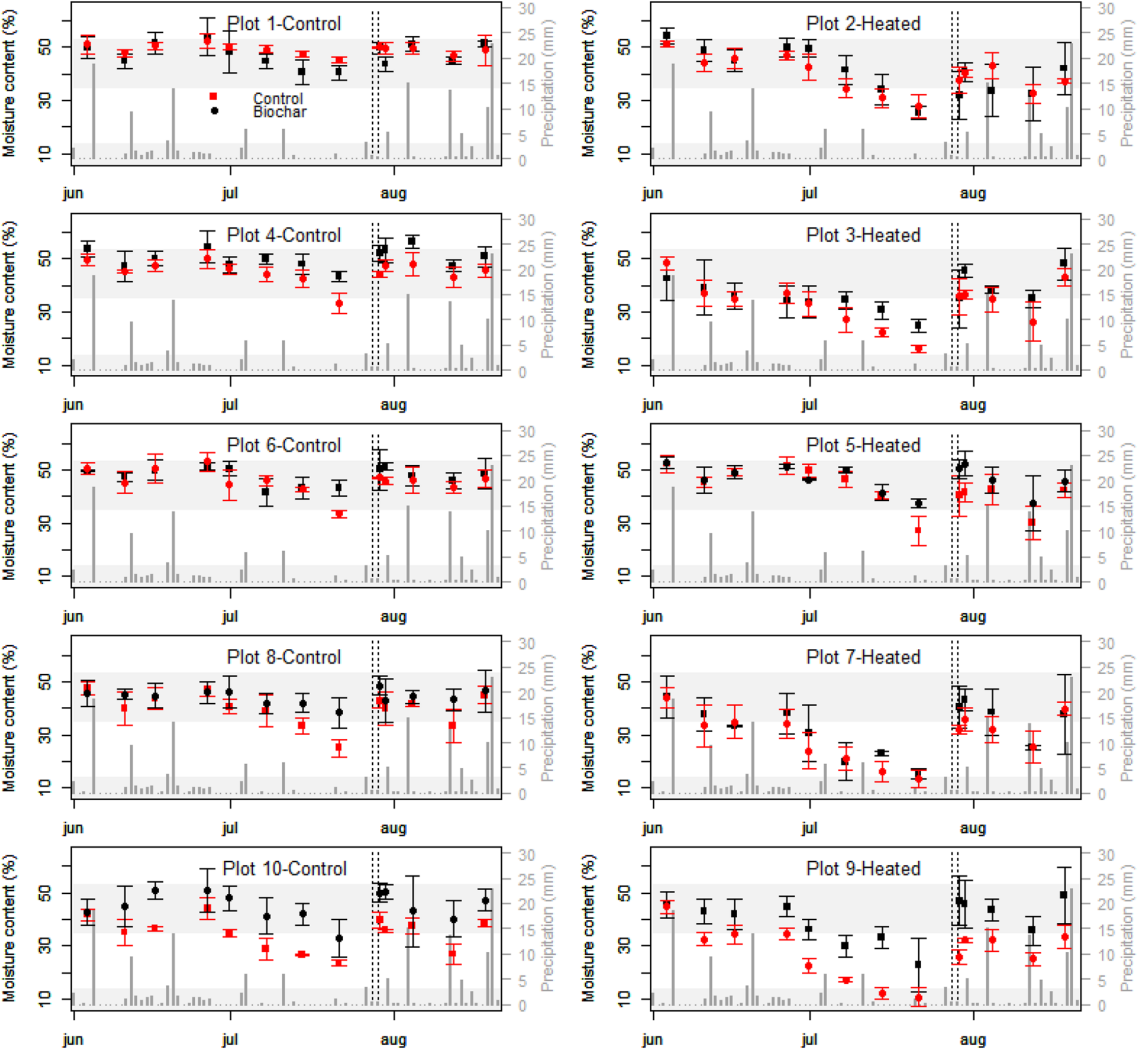
Surface soil moisture content per plot and per treatments in parallel to soil water input. Moisture data are presented by mean of average and standard deviation. Both precipitation and irrigation are displayed as bar diagrams in mm of water. Bars are plain grey for precipitation and dashed black for Irrigation. Horizontal grey zones represent, from top to bottom, the average moisture content ± standard deviation, at saturation (i.e. 43.9 ± 9.3%) and at wilting points (i.e. 6.5 ± 7.3%) for 330 silt loam soils as given by the Rosetta program developed by the USDA to estimate hydraulic parameters through pedotransfers functions [42]

Volumetric soil moisture of the surface layer averaged 40.9% ± 9.0%, had a mode at 46% and ranged between 10.6 and 56.5%, with half of the data being included between 34.9 and 47.6% (Fig. 5). Soil moisture content was directly linked to precipitation and irrigation. In June and August, abundant precipitation in quantity and frequency (i.e. respectively 60 and 73.5 cumulative mm of water for 18 days of rains) enabled volumetric moisture content to remain close to its maximum, whereas in July the reduction of the precipitation (i.e. 25.4 cumulative mm of water for 8 days of rain) induced a decrease of the soil moisture content which, was ended with the irrigation on July 28–29 (Fig. 5).

The linear mixed effect model analysis showed that warming decreased soil moisture by about 7.93% (standard error: ± 1.68) [χ^2^(1) = 8.53, p = 0.0035]. In contrast, biochar significantly increased soil moisture by 4.37% (standard errors: ± 1.26) [χ^2^(1) = 7.99, p = 0.0047].

Although TDR measurements indicated that the soil beneath the surface was always moist, hardening and crusting of bare surface soil patches were observed in the heated plots.

### Effects on vegetation

**Fig. 6:**
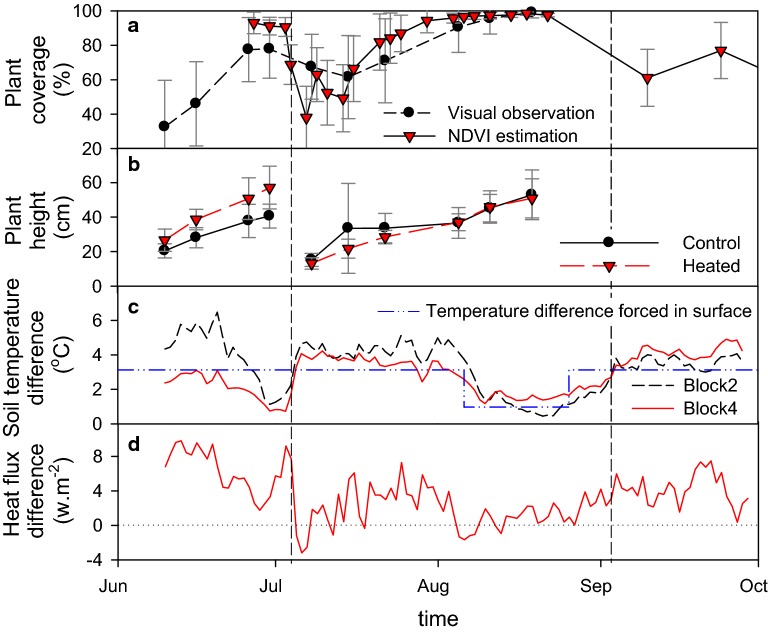
Radiative transfers in the soil-canopy system. **a** Percentage of plant coverage, **b** plant height, **c** daily average of soil temperature differences between warmed and control plot for block 2 and 4, **d** average daily heat fluxes differences between warmed and control plots. Vertical lines represent the position of the first and second harvest

Plant coverage estimations were highly variable, reflecting the patchiness of the vegetation establishment. However, both visual and NDVI-based estimation methods showed that plant coverage increased with time from 0 to ~ 80% and from ~ 60 to ~ 100% during the first and second growing periods respectively, with full coverage reached by the end of July (Fig. 6a). The small decrease of the surface covered by plants after harvest is directly explained by the reduced leaf coverage. Warming did not significantly modify the percentage of vegetation cover during the first growing period [χ^2^(1) = 4.90, p = 0.09], but significantly decreased it during the second growing period by about 30% (standard error: ± 8.2) in mid July [χ^2^(1) = 10.11, p = 0.0015]. No significant effect of biochar on percentage plant cover could be observed within the course of the first [χ^2^(1) = 0.06, p = 0.801] and the second growing periods [χ^2^(1) = 0.24, p = 0.627].

Just before the first harvest, plant height reached about 57 ± 12 cm and 41 ± 7 cm for warmed and control plots respectively, whereas 46 days after that no difference could be observed with plant height in both treatments (Fig. 6b). The linear mixed effect model analysis (Table 5) showed that warming significantly increased plant height during the first growth period by about 16.5 cm (standard error: ± 3.4) [χ^2^(1) = 8.78, p = 0.003], but not during the second growth period [χ^2^(1) = 0.23, p = 0.63]. Similarly, warming significantly increased the dry weight of the first harvest by about 22.3 g per sub-plot (standard error: ± 9.0) [χ^2^(1) = 4.73, p = 0.030], but it did not have any significant influence on the dry weight of the second harvest [χ^2^(1) = 0.23, p = 0.632]. Biochar had no significant effect on plant height or harvest dry weight.

**Table 5:** Effect of warming on the vegetation (linear mixed effect model)

Warming effect	Difference ± SE^†^	χ^2^ (1)	P-value
Height—1st growth period (cm)	16.5 ± 3.4	8.78	0.003**
Height—2nd growth period (cm)	–	0.23	0.63
Dry weight—1st Harvest (g)	22.3 ± 9.0	4.73	0.030*
Dry weight—2nd Harvest (g)	–	0.23	0.632
Plant cover—1st growth period (%)	–	4.90	0.09
Plant cover—2nd growth period (%)	30 ± 8.2	10.11	0.0015**

^†^Average difference observed between control and warmed plots

*, **Quick visual significance ranking

### Soil temperature difference per block between the heated and non-heated plots

Differences in average daily soil temperature between heated and non-heated plots in blocks 2 and 4 ranged between 6.5 and 0.5 °C and showed different trends before and after the first harvest (July 4) (Fig. 6c). Before the first harvest, temperature differences decreased with time from 6.4 to 1.1 °C and from 3.1 to 0.7 °C for blocks 2 and 4, respectively. After harvest on the contrary, temperature differences were generally between 3 and 5 °C and averaged 3.7 ± 0.8 °C (Fig. 6c) with the exception of the period where the heaters did not function properly between August 6 and 26.

### Soil heat fluxes

**Fig. 7:**
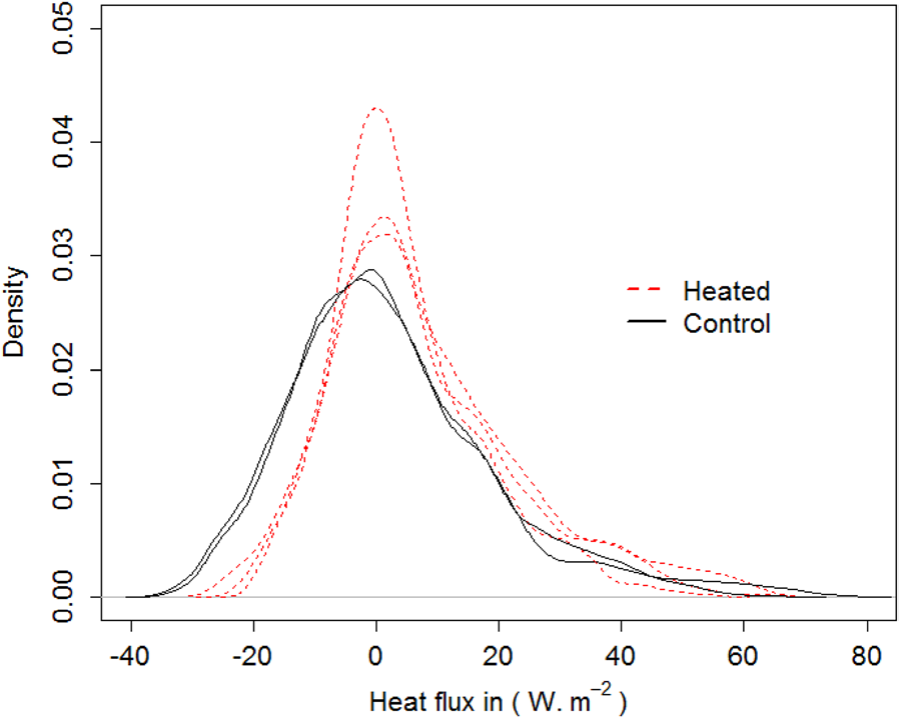
Heat flux density distribution for warming period (from Mai 6 to September 29 with the exclusion of the period where the heater did not work properly ranging from the August 4 to the August 28)

Soil heat fluxes exhibited strong diurnal variations with positive values during the day and negative values during the night, logically indicating that the soil is warming during the day and cooling at night (data not shown). From June 11 to September 28, soil heat fluxes ranged between − 33.0 and 76.5 W/m^2^, averaging 4.0 ± 14.6 W/m^2^. For 1st and 3rd quartile, the values were − 5.3 and 11.0 W/m^2^, respectively. The amplitude of diurnal variation was high in June, maximum in July, and minimum in August and September (data not shown). A heat-flux density diagram indicates that the warming effect was larger for negative heat flux, which occured principally at night, than for positive heat flux, which occured mainly during the day (Fig. 7).

Soil heat flux differences between heated and non-heated plots clearly show that warming increased the amount of energy received by the soil by an average of 4.0 ± 2.9 W/m^2^. During the period when the heaters did not function properly, the difference in heat flux averaged a non-significant 0.7 ± 1.2 W/m^2^ (Fig. 6d). Soil heat flux differences recorded between heated and non-heated plots followed the same variation patterns as those of soil temperature differences, with the exception of the first harvest date when sensors were temporarily removed from the plots (Fig. 6c, d).

**Fig. 8:**
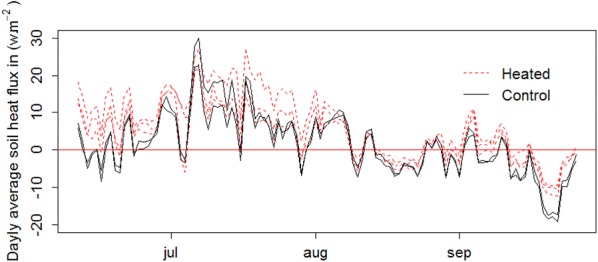
Soil heat flux

When averaged by day, heat fluxes were null and positive for the control and the warmed plots, respectively, in June, positive in July, null in August, and negative in September (Fig. 8). These results indicate that soil warming only occurred in June-July and that the warming had increased the duration of soil warming (Fig. 8).

## Discussion

### Suitability of the IR warming system

Infrared heating has been reported to generate an unrealistic vapor pressure gradient between the substomatal cavity and the atmosphere, thereby inducing enhanced evapotranspiration and drought conditions [13]. However, soil moisture monitoring showed that reduced soil water content was not such a problem for our soils, which naturally remained close to water saturation for 2 out of the 3 months of experimentation (i.e. saturation moisture content ~ 43.9% ± 9.3% for silt loam soil; upper grey band in Fig. 5). In contrast, reduced precipitation in July was responsible for decreases in soil moisture content approaching wilting point values in two out of the five heated plots (i.e. wilting point moisture content 6.5% ± 7.3% for silt loam soil; lower grey band in Fig. 5). However, irrigation promptly brought back the moisture content back up toward saturation values (Fig. 5). In a similar IR heating study, Rich et al. [29] did not supply extra water to compensate for the evapotranspiration, arguing that it would make their warming less realistic as climate warming will likely increase the soil water deficit. Similar reasoning, suggests that the small period of reduced soil moisture experienced in July might still be in the range of realistic scenario for climate warming.

In our study, the warming significantly decreased the moisture content by 7.93 ± 1.68%, which is also in the range of values observed in the literature for similar studies (e.g. 4.1 to 14% decreases in soil moisture were recorded in a semi-arid grassland and a wheat field submitted to a 1.5/3.0 °C day/night warming [12, 18]).

The effect of irrigation on the non-warmed plot will likely have little effect on the growing condition as soil moisture never departed strongly from near saturation conditions.

### Warming patterns

Our warming system successfully maintained a 3.13 °C increase in surface temperature between the warmed and the control plots of each block with high stability and accuracy. Notwithstanding that we experienced a failure of the controller system for 20 days, our results show that the system is operational provided that it undergoes regular inspection and maintenance checks.

Although the system maintained an average 3.13 °C surface temperature difference, thermal images showed that the warming was highly heterogeneous within the plot, resulting in warmer bare soil than canopy. Similar results were obtained for semi-arid grassland IR heating experiment presenting a basal vegetation cover of 50% [18]. This observed difference is likely due to reduced evaporation on a crusted soil surface combined with increased transpiration of the vegetation under IR warming [30, 31]. Similarly, bare soil patches in natural ecosystems have been reported to become warmer than the foliage of the adjacent vegetation [32–34]. Recently Kimball [25] also described a case where the emergence time for newly planted wheat was shortened by the warming treatment, with the result that heated plots with emerged wheat were actually cooler than the unheated plots that were still bare soil. He went on to propose a more complex control strategy based on canopy resistance to handle such situations when the heater treatment alters canopy architecture, physiology, albedo, or other things that can significantly alter the energy transfer characteristics of the heated plot. Reduced soil moisture of surface soil under warming, as evidenced by the linear mixed effect model, are consistent with this scenario.

In the present experiment, the soil temperature difference at 7 cm depth between the warmed and control plots of blocks 2 and 4 were as high as 6 °C (Fig. 6c), seemingly confirming the heterogeneity of the warming observed by the thermal imager that could be as high as 9.5 °C. After the first harvest, however, the soil temperature difference was rather stable but averaged + 0.6 °C higher than the warming achieved at the surface (i.e. 3.1 °C). Similar results were obtained for similar IR warming experiments carried out over ecosystems with discontinuous canopies. Higher soil warming than surface warming was observed in a semi-arid grassland with a discrepancy of + 2.1 °C and + 0.5 °C at 0.5 and 3 cm depth [18]. Similarly, in a mixed deciduous forest in the early stage of regeneration, the discrepancy was + 0.7 °C at 5 cm depth [35]. In contrast, smaller soil warming than surface warming were observed for closed canopy ecosystems, such as a Tibetan alpine ecosystem, a wheat field, and a paddy rice field [11, 12, 36, 37], suggesting that the overheating of the soil with a discontinuous plant cover is responsible for the warming discrepancy observed between soil and surfaces. A possible slight discrepancy in the depth of our temperature sensors is unlikely to generate such an effect, as the attenuation of the warming effect with depth in Arctic conditions is very progressive [4]. Differences in the amplitude of the temperature difference between block 2 and 4 (Fig. 6a, c) are most likely explained by the positions of the temperature sensors relatively to the overheated patches of bare soil.

In the literature the warming pattern produced by similar heater arrays over monospecific cultivated surfaces seems to be rather homogenous and directly linked to the homogeneity of the canopy [10, 19, 38]. In the present study, on the contrary, the thermal imager was deployed before the full establishment of the Arctic meadow that was constituted of six different plants with different height and geometry. Indeed, at the date of deployment, the plant cover in the heated plots averaged only 51 ± 22%, and was under the + 3 °C target, while bare soil patches were over it. The difficulty presented by the IR warming of a heterogeneous canopy was presented in detail by Rich et al. [29].

### Effect of warming and biochar on vegetation

IR warming had a significant effect on plant heights, yield, and plant cover, whereas biochar did not. As the development of plant cover has a direct effect on the soil warming, its study is vital to fully assess the suitability of the warming procedure. The fact that height and yield were enhanced by the warming before the first harvest, but not after, suggests that warming had significantly promoted plant emergence but not their growth rate. In that case, increased height and biomass recorded before harvest may mostly be due to an earlier start of growing period. However, considering that average air temperatures are lower in May and June (i.e. 4.5 °C and 10.5 °C, respectively) than in July and August (i.e. 14.0 °C and 11.5 °C, respectively), a preferential effect of the warming on plant growth at low ambient temperature may also not be excluded.

Slower plant colonization (i.e. the increase of plant cover) for the warmed plot compared to the control plot could be related to the observed preferential drying and crusting of bare soil under heating that would impede the emergence of tiny new plants. Alternatively, warming temperature as high as 6 °C recorded over bare soil patches may have induced dormancy in seeds [39] or also inhibited or delayed germination [23, 40]. Indeed Graae et al. [23] who submitted an Arctic tundra to a 13-day heating of the surface by 2–8 °C, showed a negative impact on seedling recruitment of several common species in Arctic ecosystem, whereas Hoyle et al. [40] showed that a 4 °C warming of an alpine soil would reduce the overall germination.

The fact that the effect of warming on plant cover only started to be significant after the first harvest seems to be directly related to the heterogeneity of the warming. Indeed, differences of warming, between bare soil and vegetation patches, must increase with increasing plant cover, as heating is increasing to compensate for plant transpiration. As a result, warming and drying of the bare soil patches should increase with time.

### Transfers of energy through the canopy

Heat-flux measurements in the soil suggested that soil warming was more effective at night when incoming solar radiation was minimum than during the day (Fig. 7). An explanation could be that a lower level of energy transfer through the canopy to the soil due to increased convection (i.e. wind) during the day. Similarly, in a boreal forest, Rich et al. [29] showed that canopy warming, as measured with IR radiometers, was easier to control and maintain during nighttime than daytime, presumably due to calmer conditions during daytime. For the same reason, Luo et al. [11] showed that in a Tibetan alpine ecosystem, the IR warming had an effect on the soil temperature at night and in mornings but not in the afternoons. The fact that the soil temperature difference between the warmed and control plots decreased with time before the first harvest in our experiment while it remained relatively stable after that is relatively difficult to explain, but it may be related to the radiation interception by the vegetation [16]. Radiation interception is a function of vegetation height and will therefore increase with time as vegetation grows, thereby inducing a reduced temperature difference between heated and control plots. Different vegetation height before the first harvest (Fig. 6b) may additionally exacerbate the phenomenon before harvest (Fig. 6c). However, the absence of decreasing soil temperature differences after the first harvest (Fig. 6c) suggests that vegetation growth and height may not be the only factors involved. Plant cover as well as the position of the temperature sensors relative to the bare soil patches may have impacted soil temperature differences observed after the first harvest. The removal and the replacement of the temperature sensors before and after the first harvest may have resulted in shifting their positions from vegetated to bare soil patches, the latter being more amenable to installing sensors. The modification of the conditions of radiation interception directly above these two sensors before and after harvest could explain stable soil temperature differences after harvest.

### Effect of biochar

Biochar did not have any significant effect on plant heights, yield, and plant cover, but significantly increased soil moisture by 4.37%. Although this value is somewhat uncertain as soil moisture sensors had to be calibrated separately for biochar amended and control plots, this last result adds up to a large body of literature showing that biochar improves soil water retention (see review [41]). The increased soil water content with biochar had no apparent effect on plant responses to the temperature treatment, suggesting that availability of soil water was not a major controlling factor in these sub-plots.

## Conclusions

One of the objectives of this work was to determine whether arrays of infra-red heaters could be used to simulate global warming in an Arctic agroecosystem. One of the requirements we had was to be able to simulate homogenous warming over large surface areas. One peculiarity of the Arctic is that global warming significantly increases the length of the growing season.

In the present experiment we had selected an Arctic meadow that was re-plowed and re-sown every 7 years, and we started the warming directly after spring snow melted to maximize the effect on growing season length. Both the increase of the growing season length and the targeted 3 °C warming were observed in our 10.5 m^2^ plots. However, when the warming was applied on bare soil (i.e. just after plowing and reseeding), differential emergence of the meadow plants impaired the homogeneity of the warming with patches of bare soil exhibiting temperatures up to 9.5 °C higher than vegetated patches. These conditions generated crusting of the bare soil surface even in near saturation conditions at 7 cm depth, which together with high local temperature increases further impacted the spatial development of the vegetal cover. This result clearly suggested that arrays of infra-red heaters are not suitable to study the effect of global warming on vegetation establishment. However, the observed buffering effect of the vegetation suggests that on years where the meadow has not been re-plowed, senescent vegetation under the snow would prevent the extreme heterogeneous warming to occur within the warmed plots.

## Additional files

**Additional file 1.** Hexagonal infrared heater array.

**Additional file 2.** Schematic representation of the IR heater arrays. Heaters represented by red dots. Heater orientation represented by red arrows. Z and θ represent heaters’ height and tilt angle respectively.

**Additional file 3.** Theoretical thermal radiation distribution uniformity over a heated plot (i.e. angle factor) as determined in Kimbal et al. (2012) for 2 different heaters height (Z) and tilt angle (θ) [8]. On the upper part of the graph intensity of thermal radiation over the heated plot is presented on the vertical axis. On the lower part of the graphic the intensity of thermal radiation is presented on a color scale ranging from red/low temperature to white/high temperature. Small dots represent heaters positions. The large black circle represent the area in which is calculated the relative range [(maximum-minimum)/mean] and the coefficient of variation CV. The left setting was selected for this experiment.

**Additional file 4.** Schematic representation of the position of the infrared radiometer (IRR) sensor relatively to the studied circular plots.

**Additional file 5.** Theoretical Field of view of the infrared radiometer (IRR) sensor for the setting presented in Additional file 4. (in blue) over the theoretical warming distribution selected for this study. The green dot represents the vertical position of the IRR.

## Abbreviations

GHG: green house gases
C: carbon
IR: infrared
OC: organic carbon
CV: coefficient of variation
IRR: infrared radiometer
TDR: time domain reflectometry
PID: proportional-integrative derivative
