# Supplementary figures and images for: Controlled infrared heating of an artic meadow: challenge in the vegetation establishment stage
Source: Plant Methods. 2019 Jan 19;15:3. doi: 10.1186/s13007-019-0387-y (PMC6339320; doi:10.1186/s13007-019-0387-y)

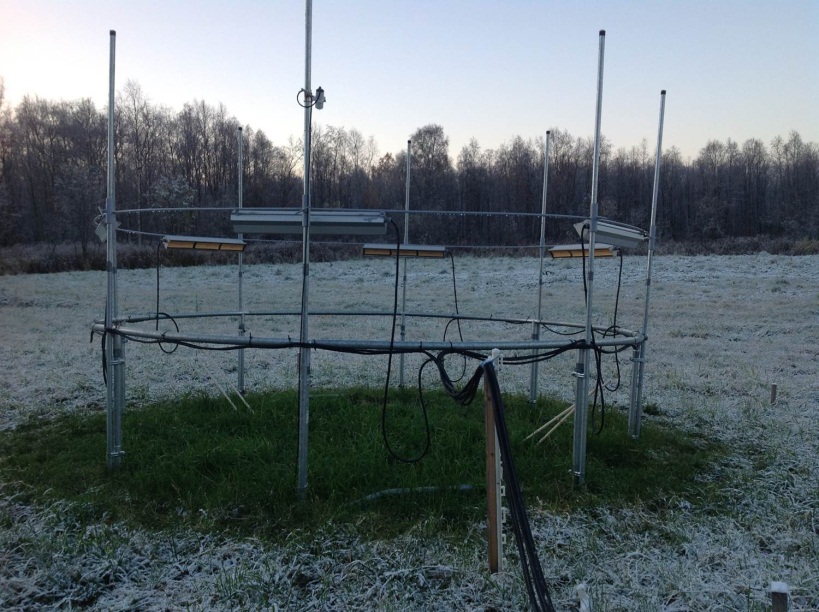

Supplement: Supplementary file 1 — Additional file 1. Hexagonal infrared heater array. [file 13007_2019_387_MOESM1_ESM.docx]

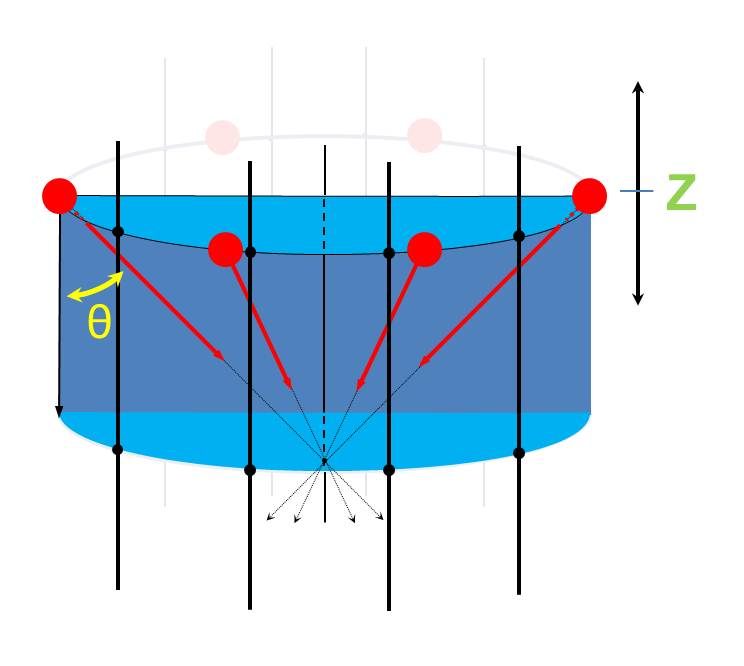

Supplement: Supplementary file 2 — Additional file 2. Schematic representation of the IR heater arrays. Heaters represented by red dots. Heater orientation represented by red arrows. Z and θ represent heaters’ height and tilt angle respectively. [file 13007_2019_387_MOESM2_ESM.docx]

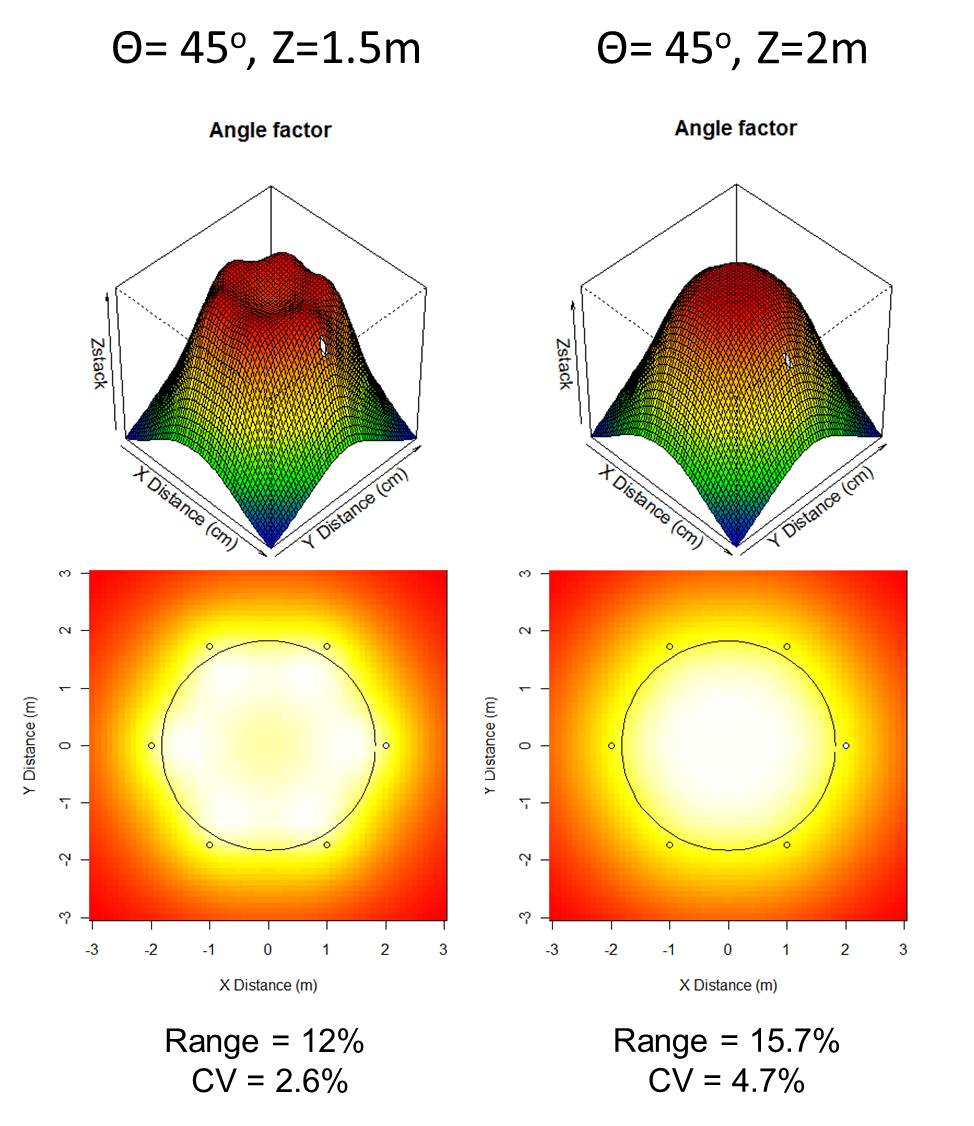

Supplement: Supplementary file 3 — Additional file 3. Theoretical thermal radiation distribution uniformity over a heated plot (i.e. angle factor) as determined in Kimbal et al. (2012) for 2 different heaters height (Z) and tilt angle (θ) [8]. On the upper part of the graph intensity of thermal radiation over the heated plot is presented on the vertical axis. On the lower part of the graphic the intensity of thermal radiation is presented on a color scale ranging from red/low temperature to white/high temperature. Small dots represent heaters positions. The large black circle represent the area in which is calculated the relative range [(maximum-minimum)/mean] and the coefficient of variation CV. The left setting was selected for this experiment. [file 13007_2019_387_MOESM3_ESM.docx]

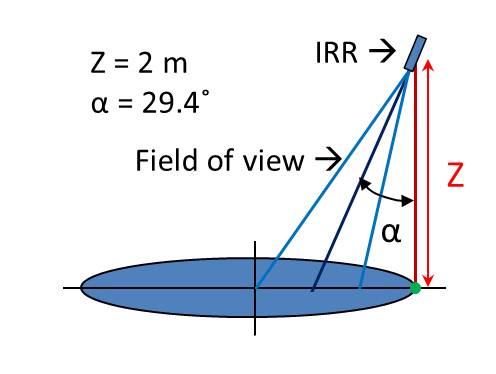

Supplement: Supplementary file 4 — Additional file 4. Schematic representation of the position of the infrared radiometer (IRR) sensor relatively to the studied circular plots. [file 13007_2019_387_MOESM4_ESM.docx]

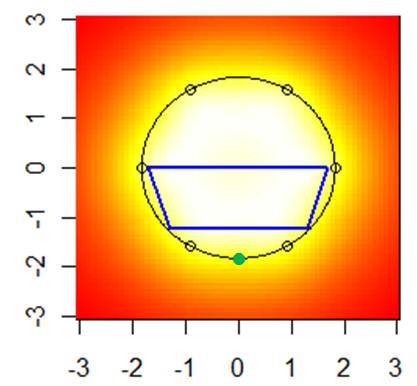

Supplement: Supplementary file 5 — Additional file 5. Theoretical Field of view of the infrared radiometer (IRR) sensor for the setting presented in Additional file 4. (in blue) over the theoretical warming distribution selected for this study. The green dot represents the vertical position of the IRR. [file 13007_2019_387_MOESM5_ESM.docx]
